# Quiescent-Interval Single-Shot Magnetic Resonance Angiography

**DOI:** 10.3390/diagnostics8040084

**Published:** 2018-12-18

**Authors:** Aman Saini, Alex Wallace, Hassan Albadawi, Sailendra Naidu, Sadeer Alzubaidi, M. Grace Knuttinen, Anshuman Panda, Rahmi Oklu

**Affiliations:** Division of Vascular and Interventional Radiology, Laboratory for Minimally Invasive Therapeutics, Mayo Clinic, Phoenix, Arizona 85054, USA; Saini.Aman@mayo.edu (A.S.); Wallace.Alex@mayo.edu (A.W.); albadawi.hassan@mayo.edu (H.A.); Naidu.Sailen@mayo.edu (S.N.); alzubaidi.sadeer@mayo.edu (S.A.); knuttinen.grace@mayo.edu (M.G.K.); panda.anshuman@mayo.edu (A.P.)

**Keywords:** quiescent-interval single-shot, non-enhanced MRA, MRA, peripheral arterial disease

## Abstract

Lower extremity peripheral arterial disease (PAD) is a chronic, debilitating disease with a significant global burden. A number of diagnostic imaging techniques exist, including computed tomography angiography (CTA) and contrast-enhanced magnetic resonance angiography (CEMRA), to aid in PAD diagnosis and subsequent treatment planning. Due to concerns of renal toxicity or nephrogenic systemic fibrosis (NSF) for iodinated and gadolinium-based contrasts, respectively, a number of non-enhanced MRA (NEMRA) protocols are being increasingly used in PAD diagnosis. These techniques, including time of flight and phase contrast MRA, have previously demonstrated poor image quality, long acquisition times, and/or susceptibility to artifacts when compared to existing contrast-enhanced techniques. In recent years, Quiescent-Interval Single-Shot (QISS) MRA has been developed to overcome these limitations in NEMRA methods, with promising results. Here, we review the various screening and diagnostic tests currently used for PAD. The various NEMRA protocols are discussed, followed by a comprehensive review of the literature on QISS MRA to date. A particular emphasis is placed on QISS MRA feasibility studies and studies comparing the diagnostic accuracy and image quality of QISS MRA versus other diagnostic imaging techniques in PAD.

## 1. Introduction

Lower extremity peripheral arterial disease (PAD) is a chronic, debilitating disease that can progress to intermittent claudication and critical limb ischemia with resulting tissue loss if left untreated. The worldwide prevalence of PAD is estimated to be between 3 to 10% of the population, and these estimates significantly increase for those over 70 years of age [1]. In the United States, approximately 8.5 million people suffer from this disease, and it is the third leading cause of atherosclerotic vascular morbidity after coronary artery disease and stroke [2]. Risk factors include smoking, diabetes, hypertension, and hyperlipidemia. The initial diagnosis can be made clinically and confirmed using imaging studies, especially in patients with claudication, signs of tissue loss, or with associated risk factors.

The ankle-brachial index (ABI) is an integral tool in the screening of patients with PAD. It is relatively quick and inexpensive, and can confirm the presence of PAD, while also providing a generalized location of stenosis. An ABI < 0.90 is diagnostic of lower extremity PAD and low ABI’s have been associated with increased risks of coronary artery disease, stroke, renal insufficiency, and all-cause mortality [3,4,5]. The ABI has a sensitivity and specificity of 91% and 86%, respectively, in detecting arterial stenosis >50% [6]. However, it is limited by its ability to characterize a narrowing, i.e., specifically, the exact location and extent of stenosis or occlusion. It can often give falsely-elevated measurements in the presence of calcified vessels in diabetics and those with end-stage renal disease [1].

Duplex ultrasound is useful for characterizing the location and extent of vascular disease, in addition to arterial hemodynamics. The sensitivity and specificity of this method for detecting >50% stenosis were shown to be 80% and 98% respectively [7]; however, the diagnostic accuracy of images can vary depending upon a number of factors including operator experience, anatomic location, irregular anatomy or extent of shadowing artifact. Evaluation of the iliac arteries is technically challenging to image with sonography, and success varies considerably with body habitus. Despite some limitations, duplex ultrasound has proven its utility in post-interventional surveillance due to its quick, non-invasive and inexpensive nature.

Angiography has long been considered the reference standard for vascular imaging and the assessment of PAD when planning interventions, given its high level of spatial resolution and real-time, dynamic nature. Digital subtraction angiography (DSA) has further increased the resolution of these images, improving the ability to accurately identify stenoses, luminal irregularities, and collateralization [8]. Physiologic information can also be gained to help assess the significance of a narrowing by measuring pressures proximal and distal to stenosis. Angiography has the added benefit of allowing for simultaneous intervention and immediately assessing the outcomes of the intervention. Despite these advantages, there are risks inherent in angiography such as puncture site hematoma, vessel injury, ionizing radiation, nephrotoxic iodinated contrast, and allergic reactions.

The advent of computed tomography angiography (CTA) has allowed for non-invasive, cost-effective means of high resolution cross sectional imaging of the vasculature. Moreover, 3D visualization of the vascular abnormalities using the CTA images helps to plan and guide interventions. The diagnostic performance of CTA in symptomatic PAD was assessed in a 2007 meta-analysis of 12 studies and 436 patients. The pooled sensitivity and specificity of this method for detecting stenoses >50% were 92% and 93%, respectively [9]. Despite the benefits of CTA, the technique still has drawbacks, which include ionizing radiation, nephrotoxic iodinated contrast, and the presence of blooming artifacts which arise from heavily calcified arteries. In patients with PAD, these artifacts have been reported to significantly decrease accuracy of identifying stenosis, particularly in the below the knee arteries [9].

Contrast-enhanced magnetic resonance angiography (CEMRA) has proven itself to be a time-efficient and accurate method for the assessment of PAD. CEMRA relies on the T1 shortening effect of gadolinium contrast, accurately diagnosing the anatomic location and extent of stenosis in PAD. In a 2007 meta-analysis, Collins et al. demonstrated that CEMRA is a more accurate diagnostic technique for the detection of significant stenosis or occlusion compared to duplex ultrasound or CTA [10]. A randomized clinical trial (ISRCTN 2671851) in 2008 comparing CEMRA, CTA, and duplex ultrasound suggested that CEMRA and CTA are more clinically useful than ultrasound in the diagnostic workup of PAD [11]. Evidence comparing the diagnostic accuracy of CEMRA and DSA is conflicting. While CEMRA does not use ionizing radiation, the higher costs, reduced availability compared to CTA, susceptibility to metal and motion artifacts, and the risk of nephrogenic systemic fibrosis in patients with advanced chronic kidney disease can be limiting [12]. Furthermore, MRI is contraindicated in select patients with cardiac pacemakers. The various screening and diagnostic tools in PAD are summarized in Table 1.

## 2. Non-Enhanced MRA Techniques

Non-enhanced magnetic resonance angiography (NEMRA) techniques have been well described, and their implementation and clinical use has increased in the past two decades with refinement of new magnetic resonance imaging hardware, software, and protocols [13]. The development of modern NEMRA techniques has been driven by the need to reduce iodinated contrast exposure in patients with poor renal function (chronic kidney disease, diabetes, atherosclerotic disease) and to avoid the risk of nephrogenic systemic fibrosis with gadolinium based contrast media. Furthermore, because NEMRA lacks both ionizing radiation and intravenous contrast, it allows for repeat examinations in the same setting. It also makes these techniques more suitable for screening and for follow-up exams. Some of the most common NEMRA techniques are summarized below.

### 2.1. Time of Flight

Time of flight (TOF) MRA is a technique that exploits the inflow effect of blood protons. The repeated radiofrequency (RF) excitation of stationary tissue adjacent to the vessel being imaged leads to its saturation, resulting in low image signal. However, the protons in blood that are flowing into an imaging slice are unsaturated, as they have not yet been subject to the repeated background RF pulses. These unsaturated spins produce high signal intensity, allowing for contrast between the bright vessel and darker stationary tissues. The inflow effect is dependent on the rate at which fresh blood enters an imaging section, which is a function of its velocity, the repetition time (TR), and cross-sectional area of the vessel [14]. TOF MRA can be acquired in two-dimensional (2D) or three-dimensional (3D) modes, with each mode providing specific advantages depending upon the vasculature being imaged. For long vascular segments that are perpendicular to the imaging plane (i.e., peripheral vasculature or the aorta), the sequential slice collection of 2D TOF allows for short imaging times and increased sensitivity to slow flow [14]. Disadvantages of 2D TOF include insensitivity to in-plane flow, particularly for tortuous vessels, which can lead to signal loss and overestimation of the degree and length of stenosis [15]. For more compact vascular segments with tortuous paths (i.e., renal or cerebral arteries), 3D TOF demonstrates excellent resolution and signal-to-noise ratio. Disadvantages of this technique include longer imaging times, limitation to short vascular segments and its continuous segment acquisition, which can lead to increased motion artifact [15]. Inflow-based NEMRA techniques are also subject to venous contamination, as the inflow of unsaturated protons from fresh venous blood into the imaging slice creates a bright signal as well; therefore, venous suppression techniques using “walking” presaturation RF pulses must be applied to the venous region upstream of the imaging slice [14]. These techniques are also subject to accelerated intra-voxel phase dispersion due to turbulent flow and signal attenuation from saturation bands [13].

### 2.2. Phase-Contrast

Phase-contrast magnetic resonance angiography (PC-MRA) generates vessel imaging by displaying the accumulated phase difference in transverse magnetization between protons in blood and those in stationary background tissues [13]. This technique relies on the subtraction of two flow-encoded images, which results in the removal of the background phase accrual. Thus, stationary tissue spins are suppressed, allowing for phase difference images [14]. Phase-contrast MRA is advantageous in that it is independent of flow direction, allowing for imaging in planes parallel to the vessels, with fewer artifacts unlike TOF MRA. However, like TOF, this technique is time-consuming and subject to intravoxel dephasing due to turbulent flow [16]. Moreover, because of its subtractive nature, PC-MRA is subject to motion artifact. Due to long acquisition times and the availability of more advanced techniques, PC-MRA is not routinely used.

### 2.3. 3D Half-Fourier Fast Spin Echo

Electrocardiographic (ECG)-gated half-Fourier fast spin echo (3D FSE) imaging, commercially known as fresh blood imaging (FBI) and NATIVE SPACE (sampling perfection with application of optimized contrasts using different flip angle evolutions), is a subtractive, cardiac phase-dependent MRA technique that relies on the contrast between fast flowing arterial blood and slow flowing venous blood. During systole, fast flowing arterial blood generates a dark signal (flow void). Conversely, during diastole, the now slow flowing arterial blood generates a bright signal, while venous blood consistently generates a bright signal due to a relatively constant flow rate throughout the cardiac cycle. Images are captured in both systole and diastole and then subtracted to generate an arteriogram. Although the acquisition of two images is required for this technique, total image time has been shown to be shorter than TOF MRA. Furthermore, 3D FSE sensitivity to slow flow makes it an ideal choice in peripheral arterial imaging, and it has demonstrated acceptable diagnostic accuracy in various studies [17,18]. Since 3D FSE is cardiac-gated, it can be sensitive to arrhythmias, patient motion, and decreased image quality due to trigger delay calibration errors [14]. This technique can also lead to over-estimation of stenosis in situations of fast or turbulent flow, often seen post-stenotically [14].

### 2.4. Balanced Steady-State Free Precession

Balanced steady-state free precession (bSSFP) is another common NCMRA technique, which uses T2/T1 weighted imaging ratios to contrast arterial blood from adjacent tissue. This sequence depicts all blood, including venous blood, within a slice; therefore, some type of venous suppression pulse is required [14]. Additionally, fat appears bright on this sequence, and when coupled with fat suppression, this technique has an excellent signal to noise (SNR) ratio [13]. Other advantages of bSSFP are the quick acquisition times and independence from flow direction as the spins are fully balanced in all three directions [19]. A limitation of this technique is the susceptibility to off-resonance banding artifacts in the presence of magnetic field inhomogeneities. Balanced SSFP has demonstrated its use in aortic and renal imaging [20].

### 2.5. Quiescent-Interval Single-Shot MRA

**Figure 1:**
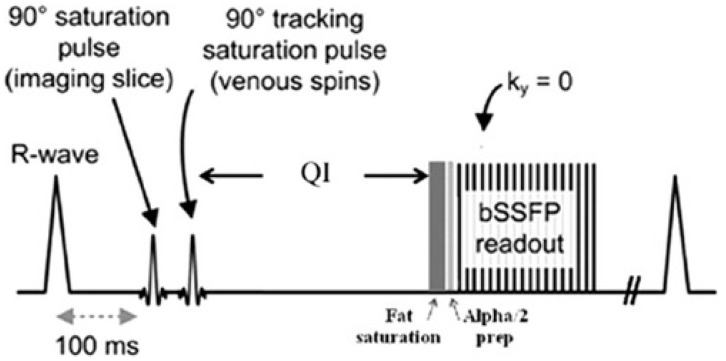
Depiction of pulse sequences in QISS MRA. Reproduced with permission from [21]. ms: millisecond; QI: Quiescent interval.

Quiescent-interval single-shot (QISS) MRA is an ECG-gated inflow based technique that has been recently developed and Food and Drug Administration approved for peripheral arterial imaging. The sequence relies on a pre-saturation RF pulse to saturate the stationary tissues in the imaging slice, followed by a venous suppression pulse. A “quiescent interval” (QI) coinciding with systole then occurs, during which there is maximum inflow of unsaturated blood into the imaging slice. A fat saturation pulse is then applied and the signal is then acquired during diastole using a 2D bSSFP sequence. The entire process is then repeated until all required images are captured [21] (Figure 1). QISS offers shorter acquisition and exam times compared to TOF MRA. Furthermore, because of its non-subtractive nature and single acquisition, image quality is less affected by motion artifacts which are often an issue in elderly patients or when imaging the abdominopelvic region. Finally, because there is no need for scout images, timing scans, or for patient-specific adjustments to imaging parameters, performing a QISS examination is a relatively straightforward and easy process [22]. Over the past 8 years, this technique has demonstrated great potential for peripheral arterial imaging in patients for whom contrast-enhanced exams may be contraindicated. The remainder of this review will focus on QISS MRA and its utility in peripheral arterial imaging, with a comprehensive review of studies examining its diagnostic accuracy, image quality, and potential applications.

## 3. Discussion

### 3.1. QISS: Technical Considerations and Early Feasibility studies

**Figure 2:**
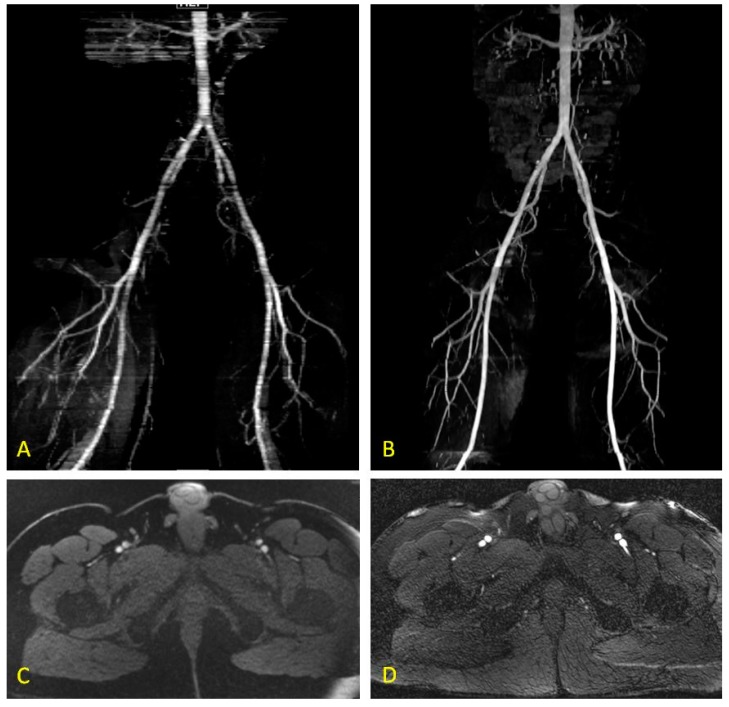
Time of flight (TOF) and QISS bilateral lower extremity MRA in the same patient. Coronal TOF MRA (**A**) and Coronal QISS MRA (**B**) of the pelvic arteries and lower extremities. Note the fine detail and improved signal throughout the distal aorta, iliac, and femoral arteries. Corresponding axial TOF MRA (**C**) and corresponding axial QISS MRA (**D**) at the level of common femoral bifurcation.

First described by Edelman and colleagues in 2010, QISS is an alternative to non-enhanced and contrast-enhanced MRA techniques, as well as DSA, for peripheral imaging in patients with PAD. It was developed in response to the long exam times, motion sensitivity, lack of reproducibility, and poor image quality of existing NEMRA techniques in the evaluation of patients with PAD. In their initial study examining the technical considerations and feasibility of QISS in peripheral MRA, Edelman et al. elucidated the technical factors associated with performing QISS at 1.5 Tesla (T), discovering the superiority of ECG gating versus pulse gating, which caused a loss of intravascular signal, and also the inferior fat suppression of full Fourier acquisition when compared with partial Fourier acquisition [21]. When comparing QISS vs. 2D TOF, the investigators found QISS MRA image quality to be superior, with reduced artifacts and shorter scan times (Figure 2). A subsequent pilot study was performed to assess the diagnostic accuracy of QISS in 8 healthy patients and 8 with documented PAD, using CEMRA as the reference standard. The sensitivity, specificity, positive predictive value (PPV) and negative predictive value (NPV) for QISS MRA in assessing clinically significant stenosis (>50%) in non-stented arterial segments were 92.2%, 94.9%, 83.9% and 97.7%, respectively. In this feasibility study, the authors demonstrated a quick and easy NEMRA technique with excellent image quality, irrespective of lower extremity disease location or severity. Moreover, unlike subtractive NEMRA methods, they showed that QISS does not need routine tailoring of the image parameters for each patient, paving the way for further investigations.

In 2011, Hodnett et al. assessed the diagnostic performance of QISS in a two center prospective trial involving 53 patients who were referred for lower extremity MRA. Using CEMRA as the reference standard, they assessed 1696 arterial segments with QISS and found that, for two readers, the sensitivities were 89.7% and 87.0% and the specificities were 96.5% and 94.6% for assessing stenosis >50% [23]. This study was the first direct comparison of QISS vs. CEMRA, and demonstrated the utility of QISS as an alternative to CEMRA in patients with PAD for whom contrast is contraindicated. Moreover, QISS lends itself to repeat examinations limited only by time on the scanner if technical difficulties were to interfere with the diagnostic quality of images—a quality that is lacking in the more invasive contrast-enhanced PAD imaging. In a separate analysis within the same year, Hodnett et al. demonstrated the diagnostic accuracy of QISS in a symptomatic diabetic population of 25 patients, again using CEMRA as a reference standard. The ABI in diabetic patients can be inaccurate due to heavily calcified, non-compressible arteries, and contrast-enhanced examinations run the risk of contrast-induced nephropathy (CIN). Therefore, the investigators examined 775 arterial segments, and the results were comparable to those in their non-diabetic study population of the same year. A subset analysis of 9 patients was also performed to assess the accuracy of QISS versus the gold standard DSA and the sensitivity, specificity, PPV and NPV for detecting clinically significant stenosis were found to be 96.2%, 96.1%, 94.4% and 97.4% [24] respectively, although these numbers should be interpreted with caution due to the small sample size. These outcomes were also mirrored by those of Klasen and colleagues, who assessed image quality and diagnostic accuracy of QISS at 1.5 Tesla (T) in a cohort of 27 PAD patients, using CEMRA as a reference standard. Subjective image quality was rated as good or excellent in 91.7% of segments using CEMRA and 89% of segments using QISS. Image quality of QISS in the distal aorta, pelvic, and femoral arteries was significantly lower than CEMRA despite the close equivalence in general. Furthermore, the degree of stenosis was overestimated with QISS in 6.3% of segments review. Despite these minor deficiencies in this small cohort, QISS was shown to have sensitivity, specificity, PPV and NPV of 98.6%, 96.0%, 88.7% and 99.6%, respectively [25].

A number of studies have also investigated the performance of QISS at 3T [26,27,28,29,30]. In a study of 12 patients with advanced PAD (Fontaine stage 2b or greater), Thierfelder et al. compared the diagnostic performance of QISS to CEMRA and found high sensitivity and specificity of 94.1% and 97.8% respectively. Similar to other QISS studies at 1.5T, the investigators also demonstrated decreased image quality of QISS in the distal aorta, pelvis and thigh [26]. QISS at 3T also showed no significant differences in the grade of stenosis in any anatomical segments when compared to CEMRA. In addition, it performed very well when discriminating between high grade stenosis and total occlusion [26], although these results should be considered with caution due to the very small cohort size. In a larger study of 25 patients, Knobloch et al. demonstrated good diagnostic accuracy for QISS at 3T, but also encountered decreased image quality in the distal aorta and pelvic region [29]. These results are contrary to the findings of Hansmann et al., who discovered that QISS at 3T demonstrated good sensitivity, but with decreased specificity when compared to CEMRA [27]. They also noted poor image quality and the prominence of motion artifacts due to long acquisition times; however, these outcomes may have been attributable to long shimming times and other technical factors [27].

### 3.2. Comparisons with Existing Non-Invasive Diagnostic Techniques

With the feasibility and diagnostic accuracy of QISS coming to light, investigators began comparisons with other non-invasive diagnostic techniques for PAD. When compared to ABI, QISS was found to have higher accuracy for detecting clinically relevant stenosis in PAD. In a study of 60 arterial segments, with CEMRA as the reference standard, the sensitivity and specificity of ABI and QISS were found to be 76% and 83%, and 96% and 92%, respectively [31]. Furthermore, significantly diseased arterial segments were concordant with CEMRA in 35% of ABI and 88% of QISS studies. In addition, 83.4% of cases for which QISS detected significant stenosis did not require additional imaging prior to revascularization [31]. These results highlight the potential of QISS as a screening exam in PAD patients, although larger, multi-center comparisons would be needed prior to clinical implementation.

When compared to CTA, QISS demonstrates excellent diagnostic accuracy and quality. In a study of 30 patients with PAD, the ability to detect clinically significant stenosis was compared between third-generation, dual-source, dual-energy CTA and 1.5T QISS MRA with DSA as the reference standard. Two readers analyzed 483 total segments, of which DSA results were also available for 410, and found the subjective image quality to be similar among the two techniques [32]. The diagnostic accuracy among the techniques was also similar; sensitivity/specificity for QISS were 84.9% and 97.2%, while for CTA they were 87.3% and 95.4%. Of note, certain stents and radiofrequency noise rendered a small portion of QISS segments non-diagnostic, whereas stenting, heavy calcifications and suboptimal opacification rendered a small portion of CTA images non-diagnostic [32]. Interestingly, the majority of segments excluded from CTA (7 of 8) due to heavy calcifications were diagnostic with QISS. Furthermore, QISS diagnostically visualized all of the segments that were excluded from CTA due to insufficient opacification [32]. These results support the use of QISS for the diagnosis of PAD, particularly in patients with diffuse calcific disease, and underscore its utility in patients for whom contrast is contraindicated.

More recently, the image quality and diagnostic accuracy of QISS at 3T was compared to CTA [33]. In 32 patients, 19 of whom underwent DSA, the investigators noted decreased image quality of QISS when compared to CTA. Conversely, the sensitivity and specificity for QISS were noted to be higher, and in heavily calcified segments these differences were statistically significant. The authors attributed the differences in image quality between the two methods to either the greater spatial resolution of CTA or motion artifacts in the longer acquisition time for QISS studies.

### 3.3. Comparison to Non-Enhanced MRA Techniques

Phantom studies have also provided great insight into the signal properties of QISS when compared to other NEMRA techniques, particularly over a range of flow velocities. Using a pulsatile flow phantom with blood-mimicking fluid, and triphasic and monophasic flow profiles, Offerman and colleagues evaluated various NEMRA techniques to examine vascular signal proximal to, within, and distal to, a 50% stenosis. Notably, QISS displayed reliable signal at high velocities in the vicinity of the 50% stenosis and best represented the morphology of the stenosis over the broadest range of velocities when compared to the other NEMRA techniques [34]. Moreover, TOF showed post-stenotic signal loss that was not observed in QISS [34]. All NEMRA methods performed similarly under monophasic and triphasic flow.

Direct comparisons of QISS to other NEMRA techniques have yielded interesting results, and in 2013, Ward et al. contrasted QISS to Native SPACE (3D FSE). In this prospective study, 20 patients referred for lower extremity MRA underwent QISS and Native SPACE at 1.5T, using CEMRA as the reference standard. Four hundred and ninety-six arterial segments were imaged, and the image quality was rated significantly higher for QISS than for Native SPACE. 24 Native SPACE images were considered non-diagnostic, whereas all QISS and CEMRA images were considered diagnostic. Although there was no significant difference in the sensitivity for detecting clinically-significant stenosis between the two methods, QISS was found to have significantly better specificity (95.6% vs. 87.3%) [35]. In the abdominal and pelvic areas, Native SPACE tended to exaggerate disease extent due to poor image quality from motion artifacts or flow dephasing [35]. In 2015, Zhang et al. compared the diagnostic performance of QISS with that of flow-sensitive dephasing (FSD) SSFP in 153 calf arterial segments of 26 patients at 1.5T. The investigators noted significantly better image quality using FSD-SSFP in the peroneal and posterior tibial arteries, and also better contrast-to-noise and signal-to-noise ratios when compared to QISS; however, both techniques produced satisfactory image quality. There were no significant differences in sensitivity and NPV in determining clinically-significant stenosis between the two methods; however, FSD-SSFP was noted to have higher specificity (99% vs. 92%) and diagnostic accuracy (98% vs. 92%) compared to QISS [36]. FSD-SSFP, however, does require scout images and other patient tailored imaging parameters, making it a lengthier and more complicated exam. In 2017, Altaha and colleagues compared the performance of QISS and 3D Turbo Spin Echo (TSE) in critical limb ischemia (CLI). Nineteen patients with CLI underwent both techniques at 1.5T, and the results were compared with DSA. Two readers evaluated 267 arterial segments and rated the image quality of QISS as good to excellent in 79.5% and 96% of segments. Surprisingly, approximately 90% of 3D TSE segments were rated to be non-diagnostic or of poor image quality [37]. The sensitivity and specificity for clinically significant stenosis for QISS in this cohort were 92% and 95% respectively. The results of this study support the use of QISS in CLI patients but should be confirmed in larger populations prior to clinical implementation. Studies comparing the diagnostic accuracy, strengths and limitations of QISS to other PAD diagnostic modalities are summarized in Table 2.

### 3.4. Technical Advancements and Alternative Applications

In addition to comparing the diagnostic performance of QISS with other NEMRA techniques, several studies have examined variations on the technical aspects of the QISS technique and their effects on diagnostic outcomes. In a 2013 study, Edelman et al. examined the possibility of accelerating QISS image acquisition by using high under sampling factors and a radial k-space trajectory. In doing so, three slice acquisitions were obtained per cardiac cycle in scan times as short as two minutes for the whole leg [38]. Moreover, the use of radial k-space allowed for the preservation of spatial resolution despite under sampling. This technique was, however, subject to striping artifact because of the differing T1 relaxation times due to multi-slice acquisition. Nonetheless, these technical modifications allow for a complete peripheral MRA in a very short amount of time, which may have important clinical ramifications in the imaging of patients who are unable to lie flat or still for extended periods of time [38]. Investigators have also examined methods for ungated QISS, which may be useful in patients with irregular heart rhythms or large body habitus. By using radial k-space trajectory with optimized azimuthal equidistant projections and a longer quiescent interval, QISS can be performed without ECG-gating, and with minimal horizontal striping [39]. This method does, however, have some potential drawbacks, including longer scan times, decreased venous suppression, and the need for off-line fat suppression [39].

Investigations into the applications of QISS outside of peripheral arterial imaging have also been performed, with initial feasibility studies showing promising results. Extracranial carotid artery QISS at 3T has demonstrated good image quality with ECG-gating and good concordance with CEMRA with respect to disease grading [40]. In a technical feasibility study of 14 patients without pulmonary embolism (PE) or occlusion, radial QISS showed quick image acquisition (2–3.5 min), good image quality in breath-hold and free-breathing versions, and good conspicuity of the pulmonary arteries as determined by three readers through the level of the segmental branches [41]. More recently, a retrospective study of 14 patients with congenital heart disease utilized QISS to visualize coronary origins with clinically-acceptable to good image quality [42]. Although further technical optimization and sufficiently powered studies are needed, QISS MRA shows exciting potential outside of peripheral arterial imaging.

## 4. Conclusions

Quiescent-Interval Single-Shot MRA has shown itself to be an accurate and robust sequence in the diagnosis of patients with suspected PAD of the lower extremity. When compared to contrast-enhanced MRA, CTA, or the more invasive angiography, it demonstrates excellent diagnostic performance, image quality, and ease of use in a number of prospective cohorts. Furthermore, the absence of nephrotoxic contrast makes QISS an ideal technique for those in whom contrast is contraindicated—a significant portion of symptomatic PAD patients. Despite its proven utility, further technical optimizations and multi-center trials are needed prior to its widespread clinical implementation. Nevertheless, it offers a non-contrast alternative to conventional imaging techniques with the potential for a wide variety of clinical applications.

## Figures and Tables

**Table 1:** Screening and Diagnostic Tools in Peripheral Arterial Disease.

Technique	Strengths	Limitations
ABI	Quick, inexpensive, highly sensitive for arterial stenosis >50%	Cannot characterize extent or exact location of stenosis/occlusion, limited accuracy in presence of highly calcified vessels
Duplex US	Quick, inexpensive, can characterize extent and location of disease, utility in post-interventional surveillance	Limited by operator experience, irregular anatomy, shadowing artifact
DSA	High resolution, provides physiologic information, allows for simultaneous intervention and assessment	Invasive, potential for vessel injury, ionizing radiation, nephrotoxic iodinated contrast use, allergic reactions
CTA	Quick, high resolution, 3D reconstructions assist in interventions, high diagnostic accuracy, cost-effective	Nephrotoxic iodinated contrast use, ionizing radiation, blooming artifact
CEMRA	High resolution, no ionizing radiation, 3D reconstructions assist in interventions, high diagnostic accuracy	Costly, reduced availability, metal and motion artifact, concern for NSF with gadolinium-based contrast
NEMRA	No iodinated or gadolinium based contrast removes concerns for renal toxicity/NSF, no ionizing radiation, allow for repeat screening/follow-up exams	Variable image quality, diagnostic accuracy and artifact susceptibility, costly, longer exam times, reduced availability

ABI = Ankle-Brachial Index, US = Ultrasound, DSA = Digital Subtraction Angiography, CTA = Computed Tomography Angiography, CEMRA = Contrast Enhanced Magnetic Resonance Angiography, NEMRA = Non-enhanced Magnetic Resonance Angiography.

**Table 2:** Summary of studies comparing the diagnostic accuracy, strengths and limitations of QISS to other PAD diagnostic modalities for clinically significant stenoses.

	ABI	CTA	CEMRA	2D TOF	3D FSE	(FSD) SSFP
**Reference**	[31]	[32]	[23]	[21]	[35]	[36]
**Sensitivity/Specificity**	QISS: 96%/92%, ABI: 76%/83%	QISS: 85%/97%, CTA: 87%/95%	QISS: 85-90%/95-97%, CEMRA: Ref. Std.	N/A	QISS: 85%*/96%, 3D FSE 87%*/87%	QISS: 93%*/92%, (FSD) SSFP: 95%*/99%
**Strengths of QISS compared to study modality**	Allows for characterization of extent/degree of stenosis	No iodinated contrast use, no radiation exposure	No gadolinium based contrast, allows for repeat examinations	No post-stenotic signal loss when compared to 2D TOF, improved image quality, reduced artifacts, shorter scan time	Increased image quality, no patient dependent changes to imaging parameters, less sensitive to motion	Does not require tailoring of imaging parameters, shorter scan time
**Limitations of QISS compared to study modality**	Costly, not readily available, longer exam times	Not readily available, certain stents can make some images non-diagnostic	Lower image quality in abdominopelvic vessels	In-plane signal loss for vessels not perpendicular to imaging slice, sensitive to static field inhomogeneities	Sensitive to static field homogeneities, in-plane signal loss for vessels not perpendicular to imaging slice	Lower image quality in peroneal and posterior tibial artery, lower diagnostic accuracy

(FSD) SSFP = Flow sensitive dephasing steady-state free precession; Ref. Std. = Reference standard; * = Not significantly different.
